# A Pilot Study of Linezolid for Early Syphilis Treatment

**DOI:** 10.1093/ofid/ofag690

**Published:** 2026-10-24

**Authors:** Ryan M Nguyen, Chrysovalantis Stafylis, Kelika A Konda, E Michael Reyes-Diaz, Kori Keith, Keith Stokes, Catherine Creticos, Candice J McNeil, Philip A Chan, James B Brock, Byron Buck, Ebony Warren, Rica M Abbott, Jennifer L Nall, Peter Salhaney, Takila Keys, Jeffrey D Klausner

**Affiliations:** Department of Population and Public Health Sciences, Keck School of Medicine, University of Southern California, Los Angeles, California, USA; Department of Population and Public Health Sciences, Keck School of Medicine, University of Southern California, Los Angeles, California, USA; Department of Population and Public Health Sciences, Keck School of Medicine, University of Southern California, Los Angeles, California, USA; Center for Interdisciplinary Studies in Sexuality, AIDS and Society, Universidad Peruana Cayetano Heredia, Lima, Peru; Department of Population and Public Health Sciences, Keck School of Medicine, University of Southern California, Los Angeles, California, USA; Open Arms Healthcare Center, Jackson, Mississippi, USA; Howard Brown Health, Chicago, Illinois, USA; Department of Medicine, Section of Infectious Diseases, Wake Forest University School of Medicine, Winston-Salem, North Carolina, USA; Open Door Health, Rhode Island Public Health Institute, Providence, Rhode Island, USA; Department of Medicine, Division of Infectious Diseases, University of Mississippi Medical Center, Jackson, Mississippi, USA; Open Arms Healthcare Center, Jackson, Mississippi, USA; Howard Brown Health, Chicago, Illinois, USA; Department of Medicine, Section of Infectious Diseases, Wake Forest University School of Medicine, Winston-Salem, North Carolina, USA; Department of Medicine, Section of Infectious Diseases, Wake Forest University School of Medicine, Winston-Salem, North Carolina, USA; Open Door Health, Rhode Island Public Health Institute, Providence, Rhode Island, USA; Department of Medicine, Division of Infectious Diseases, University of Mississippi Medical Center, Jackson, Mississippi, USA; Department of Population and Public Health Sciences, Keck School of Medicine, University of Southern California, Los Angeles, California, USA

**Keywords:** linezolid, repurposing, syphilis, treatment

## Abstract

In a study of oral linezolid among 18 patients with early syphilis, 15 showed adequate serological response by 6 months. Among 6 patients treated with benzathine penicillin G, 5 showed adequate serological response. There were no serious adverse events. Linezolid shows promise for the treatment of early syphilis.

## Teaser

- Fifteen of 18 (83.3%) of participants treated with oral linezolid 600 mg twice daily for 10 days showed adequate serological response by 6 months.
- No serious adverse events were reported.
- Larger clinical trials are necessary to evaluate efficacy.

Syphilis remains a prominent sexually transmitted disease that disproportionately affects men who have sex with men, those with human immunodeficiency virus infection, and has increasingly impacted pregnant women [1]. In the United States, in 2024, 186,301 new syphilis cases and 3941 new cases of congenital syphilis were reported [2].

The Centers for Disease Control and Prevention and the World Health Organization recommend a single 2.4 million units dose of intramuscular benzathine penicillin G for the treatment of early syphilis [3]. In the United States, recurring benzathine penicillin G shortages have been reported intermittently since 2016, with the most recent shortage starting in 2023 and projected to persist through the end of 2027 [4]. The penicillin crisis calls for the identification of safe, readily available treatment alternatives [5–7].

Linezolid is an FDA-approved, orally administered antibiotic with a favorable pharmacokinetic and safety profile. It is available in generic form and appears on the World Health Organization Essential Medicines list [8]. Linezolid has been used clinically for skin and soft tissue infections, pneumonia, vancomycin-resistant enterococcal infections, and drug-resistant tuberculosis [9]. Although generally safe, the most common adverse effects of linezolid treatment, such as decreased blood cell counts and neuropathy, are associated with long-term use (>4 weeks) [10]. Linezolid reversibly inhibits monoamine oxidase and can cause serotonin syndrome (<5%) in patients receiving certain medications for depression or anxiety. Due to a lack of well-controlled studies in pregnant women, linezolid should be used during pregnancy only if the potential benefit justifies the potential risk to the fetus [10].

Linezolid is highly effective against *Treponema pallidum* in vitro and in the animal model. Haynes et al (2021) [11] demonstrated that rabbits infected with *T. pallidum* and treated with linezolid showed rapid healing of lesions, absence of seroconversion, and no abnormal cerebrospinal fluid findings. Utilizing the SS14 strain of *T. pallidum*, the researchers determined that the in vitro minimum inhibitory concentration (MIC) for linezolid was 0.5 μg/mL.

A recent multicenter, prospective, open-label, non-inferiority, randomized controlled trial assessing oral linezolid 600 mg, once per day for 5 days, compared to one intramuscular injection of benzathine penicillin G 2.4 million units showed that treatment success was substantially lower in the linezolid group (70%) in comparison to 100% in the penicillin group [12]. Due to the significant difference in treatment outcomes, the trial was halted early. Post hoc analyses, however, suggested that a regimen of 600 mg, twice daily, for 10 days showed a probability of treatment success of 99%. Based on prior pharmacological and microbiological data, a dose of 600 mg twice daily at steady state yields a minimum and a maximum serum concentration that are 12 and 42 times greater than the MIC, respectively, ensuring adequate antimicrobial inhibitory concentrations [13]. Considering that linezolid exhibits time-dependent killing and that *T. pallidum* is a slow-growing bacterium, the ideal dosing regimen should maximize the exposure of drug over time. Thus, longer treatment duration and more frequent dosing are logical.

Given the favorable pharmacokinetic and preclinical profile of linezolid against *T. pallidum*, we assessed whether a 10-day treatment regimen of oral linezolid 600 mg twice daily would be effective in the treatment of early syphilis.

## METHODS

We conducted a randomized, noncomparative pilot study utilizing an experimental group and a contemporaneous control group aiming to collect a preliminary estimate of the serological response to linezolid. Considering the established serological response to penicillin, a contemporaneous group with participants receiving penicillin provided a potential comparison.

### Participants

We recruited 25 individual patients diagnosed with primary, secondary, or early latent syphilis from 5 sexual health clinics in the United States. The clinics were located in Jackson, Mississippi; Chicago, Illinois; Greensboro, North Carolina; and Providence, Rhode Island. Study activities occurred between October 2023 and August 2026.

Potentially eligible clinic patients were informed about the study, screened, and enrolled with informed consent. Table 1 shows the inclusion and exclusion criteria. Upon enrollment, we collected basic demographic information and medical history. For people with HIV infection, we collected the most recent CD4 T cell count and HIV viral load from the medical record. A study clinician conducted a brief, focused sexual and medical history and clinical examination looking for signs of syphilis, including a brief screening examination for neurosyphilis. Participants with potential signs and symptoms of neurosyphilis were excluded and referred for additional evaluation. Finally, we reviewed previous testing and syphilis treatment history by clinic medical record review or with the local Department of Public Health to stage participants appropriately [3].

**Table 1.** Inclusion and Exclusion Criteria

Inclusion Criteria	Exclusion Criteria
Diagnosed cases of primary, secondary, or early latent syphilis with RPR titer ≥1:8 within 3 wk prior to enrollment 18 y of age or older. Able to provide informed consent/assent. Individuals with HIV infection must be on treatment for HIV infection and virologically suppressed (viral load <200 copies/mL) or have a CD4 count ≥350 cells/mm^3^ as of their most recent test prior to enrollment.	Pregnancy or a positive pregnancy test on the day of enrollment. Patients showing signs and symptoms of neurosyphilis. Participants who receive certain psychotropic medications, eg, MAO inhibitors, SNRIs, SSRIs^a^. Serofast RPR titer, defined as a persistently positive RPR titer without more than a 4-fold (2-titer level) changes for 12 mo or greater. Recent (within the past 7 d) or concomitant antimicrobial therapy with activity against syphilis, namely azithromycin, tetracycline, doxycycline, cefixime, ceftriaxone, or other beta-lactam antibiotics (eg, amoxicillin). Individuals with HIV infection who report HIV treatment interruption for more than 4 wk since their most recent viral load or CD4 test. Individuals with known myelosuppressive conditions or receiving myelosuppressive medication. Individuals weighing 150 kg (330 lbs) or more. Individuals with known end-stage renal disease, for example, individuals in hemodialysis. Self-reported allergy to linezolid or penicillin. Unwilling or unable to attend follow-up visits.

Abbreviations: HIV, human immunodeficiency virus; RPR, rapid plasma reagin; MAO, monoamine oxidase; SNRI, serotonin-norepinephrine reuptake inhibitors; SSRI, selective serotonin reuptake inhibitors.

^a^Participants receiving monoamine oxidase inhibitors (*nonselective: isocarboxazid, linezolid, tedizolid, phenelzine, and tranylcypromine, MAO-A inhibitors: methylene blue, moclobemide, MAO-B inhibitors: rasagiline, safinamide, and selegiline*), serotonin-norepinephrine reuptake inhibitors (*desvenlafaxine, duloxetine, levomilnacipran, milnacipran, and venlafaxine*), and selective serotonin reuptake inhibitors (*citalopram, escitalopram, fluoxetine, fluvoxamine, paroxetine, and sertraline*) were excluded from the study.

All participants were required to return for 3- and 6-month follow-up visits. During follow-up, participants were asked about new symptoms and signs of syphilis and neurosyphilis, antibiotic use since last visit with focus on azithromycin, tetracycline, doxycycline, ceftriaxone, or other beta-lactam antibiotics, condom use, and number of sex partners since last visit. If necessary, a study clinician would conduct a new clinical evaluation for new or persistent signs and symptoms of syphilis. Those receiving linezolid had an additional 5-day and 10-day telephone check-ins to monitor adherence and adverse events, as well as a 1-month in-person visit for safety monitoring to detect early failure of linezolid (ie, a 4-fold or greater rapid plasma reagin (RPR) titer increase, emergence of new symptoms, or persistence of symptoms).

### Laboratory Testing

Participants provided a serum blood sample upon enrollment and at the 3- and 6-month visits. An additional blood sample was collected for those in the linezolid group during the 1-month visit. Syphilis testing included the nontreponemal RPR test and RPR titer (Gold Standard Diagnostics, USA; Arlington Scientific RPR Card Test, USA; Fisher Healthcare^TM^ SURE-VUE^TM^ RPR Card Test) and treponemal chemiluminescence immunoassay (DiaSorin LIAISON® Treponema Assay, USA) and chemiluminescent microparticle immunoassay (Abbott Alinity i Syphilis TP, USA). The same RPR test was performed in participants at enrollment and follow-up.

### Randomization and Interventions

We randomly assigned participants to receive either the standard-of-care treatment (benzathine penicillin G) or linezolid, with a 1:3 allocation, respectively. To ensure unbiased randomization, participants were randomized using REDCap (Research Electronic Data Capture). Those in the linezolid arm received linezolid 600 mg orally, twice daily, for 10 days (Zyvox, Pfizer USA, or available generic formulation). Those assigned to penicillin received a single dose of benzathine penicillin G 2.4 million units, intramuscularly.

### Sample Size Considerations and Data Analysis

The primary outcome was defined as a 4-fold or greater decrease in the serum RPR titer by 6 months from the titer measured on the day of treatment initiation (referred henceforth as “adequate serologic response”). Published estimates of penicillin effectiveness for early syphilis treatment vary, with some studies reporting 6-month serologic response rates around 80% [14]. To estimate the necessary sample size, we assumed that a 20% treatment failure rate would be clinically significant. With 18 participants in the linezolid treatment arm and a failure rate of 22% (4/18 failures), we estimated that the true failure rate was between 10% and 47% (95% confidence interval calculated using the exact binomial method). Treatment-associated adverse events are presented descriptively by treatment allocation. Descriptive analyses were conducted, and data were summarized as counts and percentages.

### Human Subject Considerations

The study was reviewed by Advarra IRB (Pro#00069522). The study is registered at ClinicalTrials.gov (NCT05548426) and was conducted under an Investigational New Drug application (FDA Research IND #164711). The study was overseen by a Data Safety Monitoring Board comprising a biostatistician with expertise in clinical trials, 3 clinicians, and public health experts. The Data Safety Monitoring Board met annually to review progress and safety data.

## RESULTS

### Participants

In total, 25 participants were enrolled: 18 were randomized to linezolid, 6 to penicillin, and 1 was withdrawn after enrollment, as past records review reclassified the participant as serofast (Figure 1). Study enrollment by site is shown in Supplementary materials (Supplementary Figure 1). Participant demographic characteristics are shown in Table 2.

**Figure 1.**
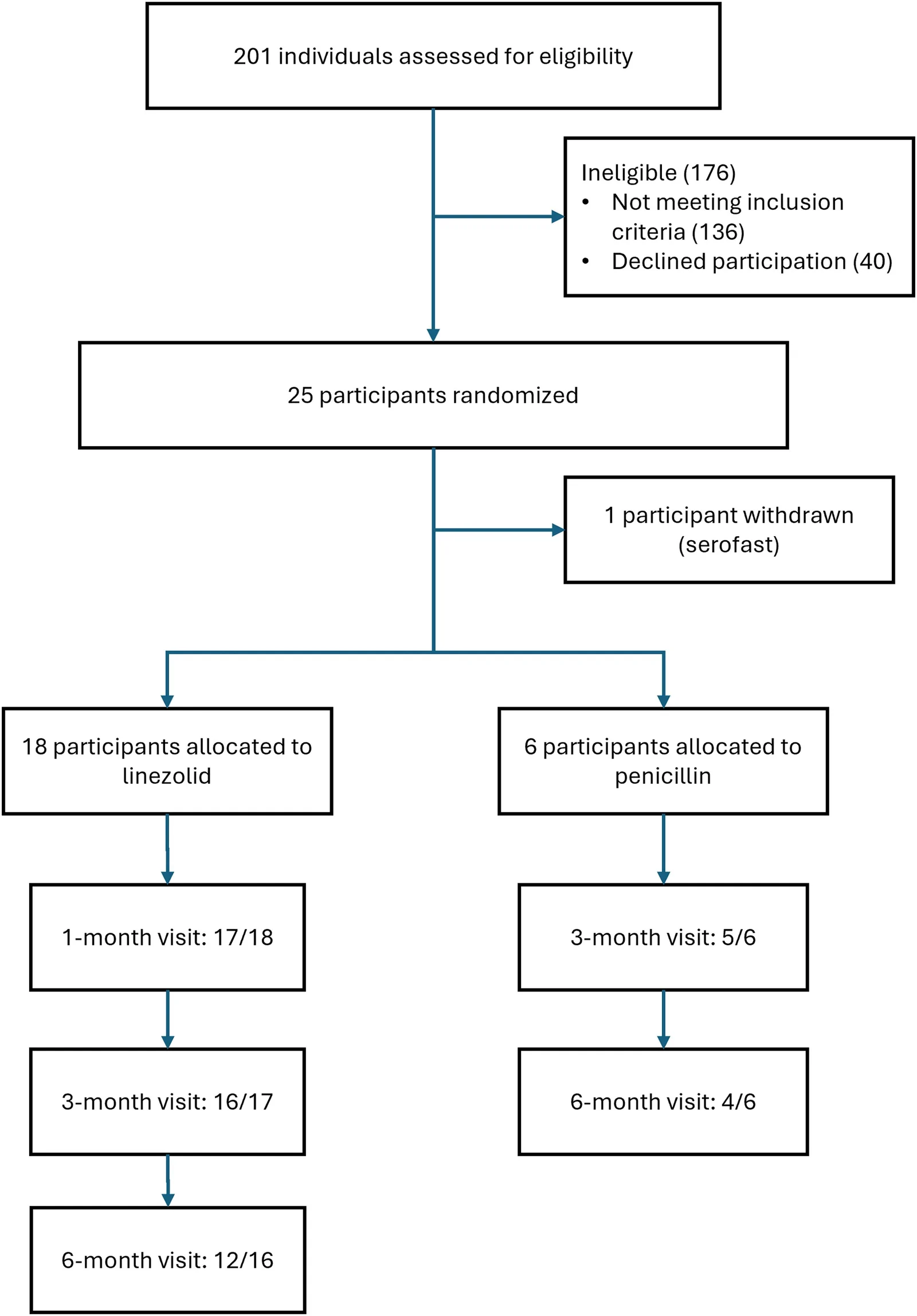
Screening, enrollment, and randomization CONSORT diagram.

**Table 2.** Baseline Characteristics of Study Participants (N = 24) by Treatment Group

	Linezolid (n = 18)		Benzathine Penicillin G (n = 6)	
Characteristic	N	%	N	%
**Male sex**	18	100	6	100
**Age**, years, median (IQR)	31.5	(27–42)	31	(24–45)
**Hispanic or Latino Ethnicity**	4	22	2	33
**Race**	…	…	…	…
White	4	22	3	50
Black or African American	11	61	3	50
Other	1	6	0	0
Declined to answer	2	11	0	0
**HIV infection (n** **=** **10)**	7	39	3	50
**On antiretroviral therapy**	7	100	3	100
**CD4 count,** cells/µL, median (IQR)	567	(464–642)	837	(633–882)
**Stage of syphilis**	…	…	…	…
Primary	2	11	0	0
Secondary	5	28	1	17
Early latent	11	61	5	83
**RPR titer upon enrollment**	…	…	…	…
1:4	0	0	1	16.7
1:8	4	22	1	16.7
1:16	2	11	3	50
1:32	7	39	1	16.7
1:128	4	22	0	0
1:256	1	6	0	0

Reported symptoms on enrollment among those reporting symptoms^a^	N = 7	…	N = 1	…
Genitourinary symptoms	4	57	0	0
Oral lesions	1	14	0	0
Anal lesions	3	43	0	0
Neck lymph nodes	1	14	1	100
Inguinal lymph nodes	4	57	0	0
Skin rash	2	28	1	100
**Enrollment site**	…	…	…	…
Howard Brown	5	28	3	50
Open Arms	8	44	2	33
Wake Forest	5	28	1	17

Data are presented as No. (%) unless otherwise indicated.

Abbreviations: HIV, human immunodeficiency virus; IQR, interquartile range.

^a^Participants may present with more than one symptom.

### Efficacy of Linezolid and Penicillin

By 6 months, an adequate serologic response was achieved by 15 (83%) of 18 in the linezolid treatment arm and 5 (83%) of the 6 participants in the penicillin treatment arm. Among the linezolid participants, 11/18 achieved a 4-fold or greater decrease by the 3-month visit, 4 participants achieved a 4-fold or greater decrease by the 6-month visit, and 3 participants did not. Among the penicillin participants, 5/6 achieved a 4-fold or greater decrease by the 3-month visit, and 1 participant was lost to follow-up.

There were 3 cases of inadequate serologic nonresponse recorded among participants in the linezolid arm summarized in Table 3. The first case exhibited a notable titer increase between enrollment and their 1-month visit and was retreated at that visit with 2.4 million units of benzathine penicillin G. The other 2 cases failed to demonstrate a 4-fold serologic titer decline by 6 months. Per protocol, both were treated with benzathine penicillin G at the 6-month visit. Self-reported adherence to the 10-day linezolid treatment regimen was 100% among all participants.

**Table 3.** Study Participants With Inadequate Serologic Response by 6 Months After Enrollment

Demographics	Syphilis Stage	RPR Titer			
		Enrollment	1-Month Visit	3-Month Visit	6-Month Visit
Male, 43 y old (linezolid)	Early latent	1:128	1:512	Withdrawn	Withdrawn
			2 sex partners		
Male, 41 y old, HIV-infected (linezolid)	Early latent	1:8	1:8	1:16	1:8
CD4 count: 567				1 sex partner	
Viral load: <20					
Male, 26 y old, HIV-infected (linezolid)	Early latent	1:16	1:8	1:8	1:8
					2 sex partners
CD4 count: 567					
Viral load: <20					
Male, 45 y old, HIV-infected (penicillin)	Early latent	1:16	-	Blood specimen not collected	Missed appointment
CD4 count: 429					
Viral load: 7960					

No serious adverse events were reported during the study. One linezolid participant reported night sweats during the treatment period, and another reported a yeast infection. Both events were addressed after consultation with the local study staff.

## DISCUSSION

In this pilot study, 15 participants with early syphilis treated with oral linezolid 600 mg twice daily achieved an adequate serological response by 6 months. Two showed a 2-fold titer decline at 6 months, and 1 showed an early titer increase. The 2 cases with only a 2-fold RPR titer decline by 6 months likely reflect the need for a longer follow-up period rather than a “true” treatment failure. The participant with the increasing titer is a case requiring further evaluation. The participant reported 2 sex partners between enrollment and the scheduled 1-month visit, which could potentially indicate reexposure to syphilis, making reinfection a possibility.

Our goal was to exclude lack of adequate serologic response early given the serious consequences of untreated syphilis. Additionally, our aim was to collect preliminary data on the frequency of an early serological response and safety, which can inform future clinical studies. Our hypothesis was that a twice-daily dosing regimen of linezolid would demonstrate an adequate serologic response in the treatment of early syphilis. Indeed, the point estimate is higher than the TrepAB study (70%) [12], although direct comparison is not appropriate because the studies evaluated different linezolid regimens. Simulated treatment success of a twice-daily regimen, arising from data from the same study, shows a potentially high success rate (99%), but verification of these simulations in humans is required.

Our study had certain limitations. This study was designed as a noncomparative pilot study with an experimental group and a contemporaneous control group. The study was not designed or adequately powered to show differences between the 2 groups, and we are unable to explore chance imbalance in patient characteristics that might have influenced the observed treatment response. Thus, the small sample size reduced the precision of the estimates. Measures of adherence, adverse events, and antibiotic use are self-reported, which may introduce bias. Additionally, the requirement for a baseline RPR titer of ≥1:8 may have selected for participants more likely to achieve the prespecified 4-fold decline in RPR titer, potentially limiting the generalizability of the observed response proportion. Study enrollment started before the introduction of doxycycline prophylaxis; however, no participants reported any use during the follow-up period. Additionally, all participants were male. Although there is no clinical reason to believe that linezolid has a different efficacy in females, future studies should include females. Once safety and pharmacokinetics are better defined in pregnant women, pregnant women should also be considered for study.

In summary, we found that 10 days of twice-daily oral linezolid resulted in an adequate serological response by 6 months after treatment among participants with early syphilis. Further, larger trials are warranted.

## Supplementary Material

ofag690_Supplementary_Data

## Supplementary Data

Supplementary materials are available at *Open Forum Infectious Diseases* online. Consisting of data provided by the authors to benefit the reader, the posted materials are not copyedited and are the sole responsibility of the authors, so questions or comments should be addressed to the corresponding author.
