# Supplementary material for: A Pilot Study of Linezolid for Early Syphilis Treatment
Source: Open Forum Infect Dis. 2026 Oct 24;13(11):ofag690. doi: 10.1093/ofid/ofag690 (PMC13728569; doi:10.1093/ofid/ofag690)
Supplement: Supplementary file 1 [file ofag690_supplementary_data.docx]

**Supplementary Figure 1. Study Enrollment by site.**
